# A Rare Case of Interdigitating Dendritic Cell Sarcoma in the Nasal Cavity

**DOI:** 10.1155/2013/913157

**Published:** 2013-04-21

**Authors:** Eun Jung Lee, Dong Woo Hyun, Hyung-Ju Cho, Jeung-Gweon Lee

**Affiliations:** ^1^Department of Otolaryngology-Head and Neck Surgery, Yonsei University College of Medicine, 250 Seongsanno, Seodaemun-gu, Seoul 120-752, Republic of Korea; ^2^Airway Mucus Institute, Yonsei University College of Medicine, Seoul 120-752, Republic of Korea

## Abstract

Interdigitating dendritic cell sarcoma (IDCS) is an extremely rare neoplasm that mainly arises from the lymphoid tissues of the immune system. Although this neoplasm typically occurs anywhere along the lymph nodes, it can also be found at extranodal sites, especially in the head and neck. We experienced a rare case of extranodal IDCS in the nasal cavity, a location that has not been previously reported. A 73-year-old woman presented with a polyp-like mass in the nasal cavity and underwent endoscopic sinus surgery. A histologic study confirmed the mass as IDCS by immunohistochemistry with S-100 antibody, and postoperative adjuvant radiotherapy was administered. Although the incidence is extremely rare, this case suggests that extranodal IDCS should be considered in the differential diagnosis of nasal cavity masses.

## 1. Introduction

Tumors arising from dendritic cells, such as follicular dendritic cell sarcoma/tumor (FDCS) and interdigitating dendritic cell sarcoma/tumor (IDCS) (WHO), are extremely rare [1, 2]. Although most dendritic cell sarcomas arise in the lymph nodes of the cervical, mediastinal, or axillary regions, approximately one-third of the cases involve extranodal sites such as the spleen, small intestine, skin, testis, ovary, urinary bladder, and tonsils [2]. Among extranodal sites, the occurrence in the head and neck area is especially low, and only eight cases in oral cavity have been reported [3–6]. We experienced a case of IDCS of the nasal cavity that was successfully treated by endoscopic surgery with postoperative radiotherapy. Immunohistochemistry with S-100, which is an interdigitating dendritic cell marker, is essential for the confirmation of diagnosis. Although the incidence is extremely rare in the head and neck, the possibility of extranodal IDCS warrants full consideration upon differential diagnosis.

## 2. Case Report

**Figure 1:**
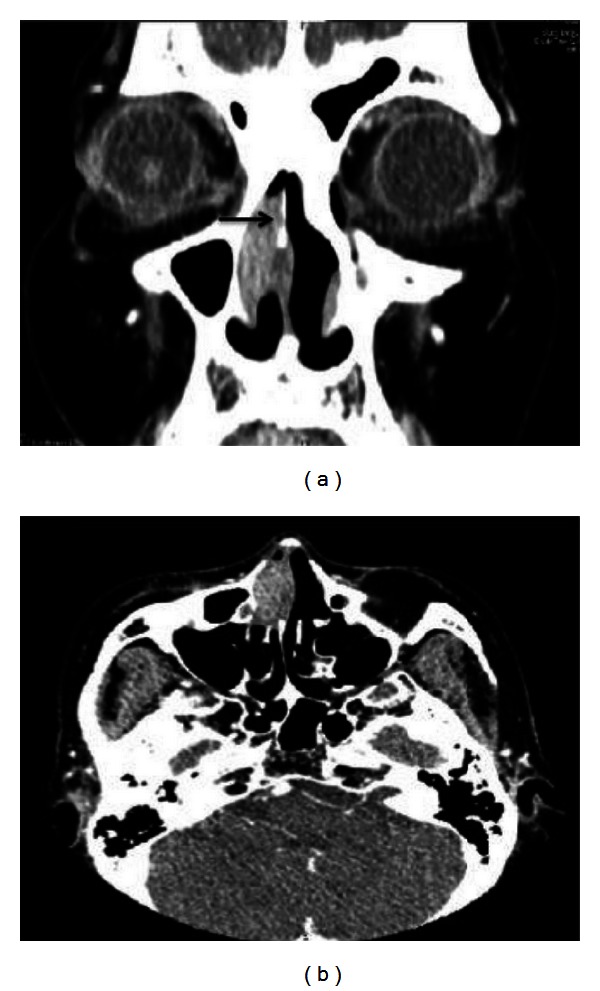
Coronal (a) and axial (b) images of paranasal sinus computed tomography. The mass is heterogeneously iso-intense between the nasal septum and lateral nasal wall, with erosion of septal bone (arrow).

**Figure 2:**
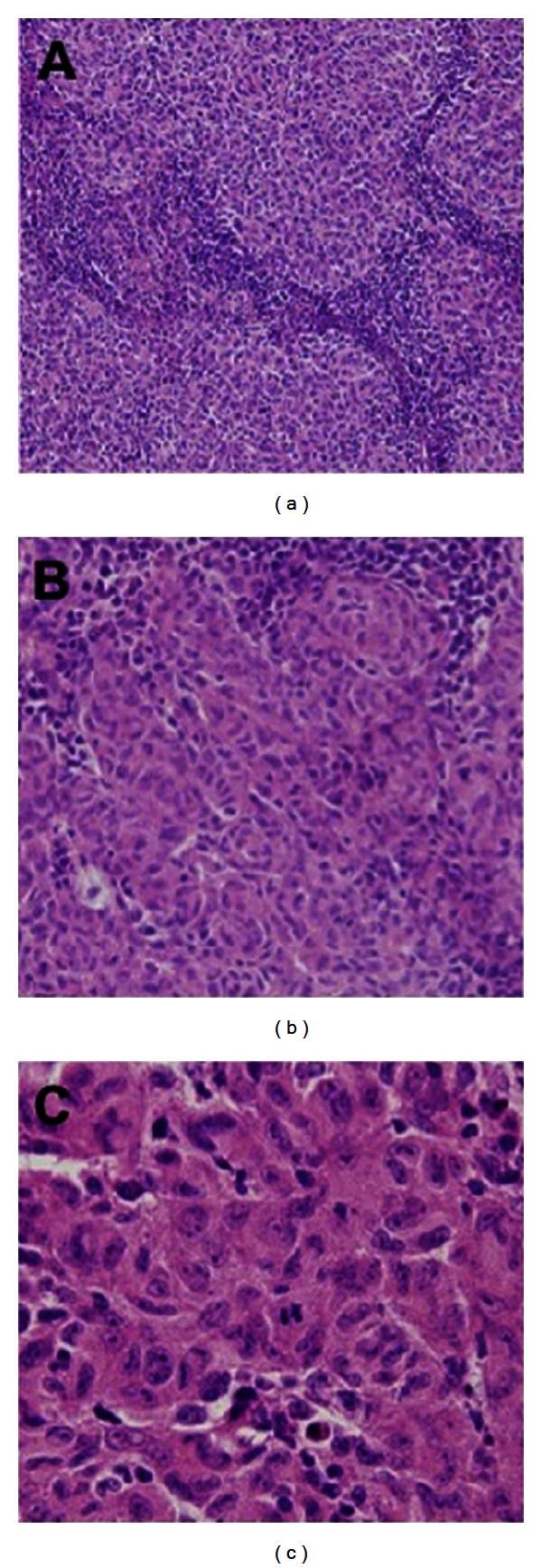
Histopathological aspects of interdigitating dendritic cell sarcoma revealing a growth pattern by hematoxylin and eosin staining. (a) Spindle-shaped cells make up the entire lesion, with small lymphocytes (×100). (b) A vaguely whorled growth pattern with abundant cytoplasm and empty nuclei (×200). (c) Spindle-shaped cells are distributed in different patterns: storiform, whorled, and occasionally palisade (×400).

**Figure 3:**
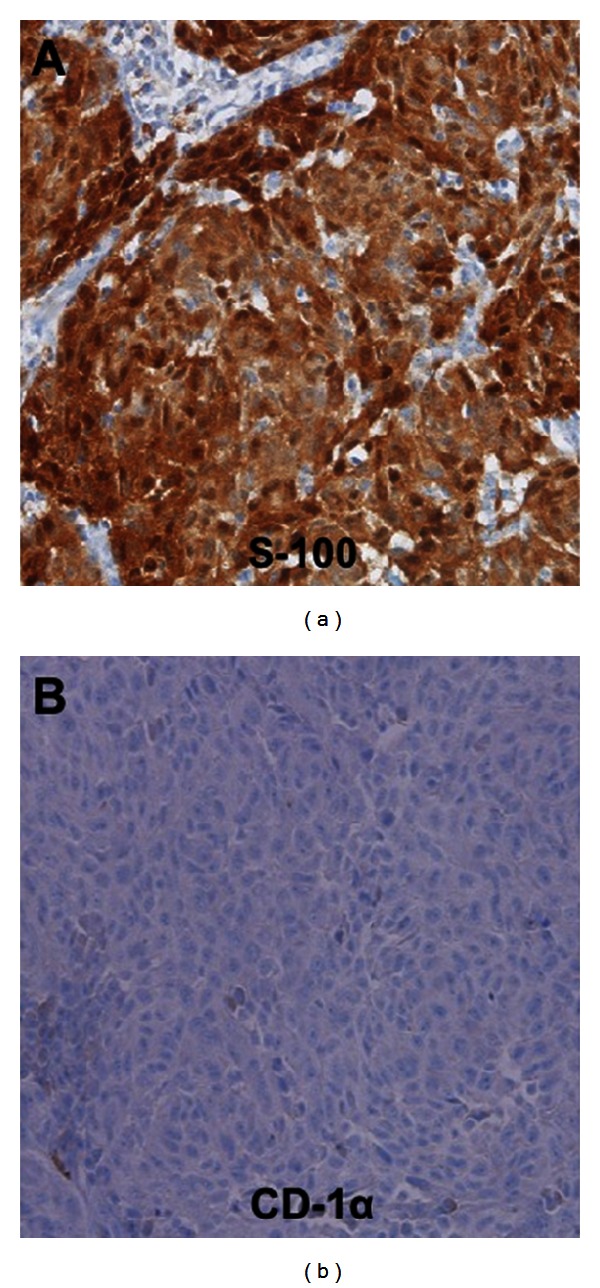
Immunohistochemical staining and S-100 were positive (a), but CD-1*α* was negative (b).

A 73-year-old woman visited our clinic and presented with a nasal obstruction and frequent blood-tinged crust that had persisted for 4 months. Initial endoscopic findings revealed a soft and fungating mass, which appeared as a nasal polyp in the middle meatus of the right nasal cavity. The mass was partially attached to the septum and was more than 1 cm long in its greatest dimension. Although it had no bleeding tendencies, hyperemic mucosa and some blood clots were noted around the mass. Paranasal sinus computed tomography (PNS CT) revealed a 2.2 cm polyp in the right middle meatus without sinusitis (Figure 1(a)). The mass had well-defined margins that were heterogeneously iso-intense on PNS CT. On axial images, the anterior cortex of the maxilla was slightly expanded with surrounding bony erosion (Figure 1(b)). It originated from the nasal septum and occupied the middle meatus. The mass was completely resected during endoscopic sinus surgery with the septal mucosa, but the septal bone and cartilage were preserved. Macroscopically, the specimen was relatively circumscribed and consisted of a single pinkish soft tissue, measuring 2.2 × 1.5 × 0.7 cm. Microscopically, the lymph nodes were diffusely effaced by a proliferation of medium to large spindle-shaped cells in a vaguely whorled growth pattern. The individual neoplastic cells had finger-like projections of cytoplasm and bizarre nuclear shapes with irregular nuclear membranes (Figure 2). The mass was confirmed as IDCS by immunohistochemistry with S-100 positive spindle cells of nodular proliferation (Figure 3). However it was negative for CD-1*α*, CD21, EMA, and D2-40. Postoperative MRI showed no residual tumor or significantly enlarged cervical lymph nodes. Adjuvant radiotherapy was administered for 2 months at 59.4 Gy at 1.8 Gy per fraction to the tumor bed area. The patient had xerostomia induced by radiation but this was tolerable. Followup PNS MRI or PET-CT revealed no abnormal FDG uptake, suggesting that there was no tumor recurrence or distant metastasis. The patient has been disease-free with no evidence of recurrence after 1.8-year followup.

## 3. Discussion

Dendritic cells are antigen-presenting cells that are heterogeneous groups of nonlymphoid and nonphagocytic immune accessory cells, which are present in lymphoid or nonlymphoid organs. Four types of dendritic cell exist in lymph nodes: follicular, interdigitating, Langerhans, and histiocytic/fibroblastic cells [2]. Among these, tumors, which are composed of interdigitating or follicular dendritic cells, are exceedingly rare. Sarcoma is also rare, and most sarcomas involve the lymph nodes. Few involve extranodal sites such as the oral cavity [3–6] or other regions of the head and neck [7–11]. 

The diagnosis of IDCS is confirmed histologically by staining with hematoxylin and eosin as well as various antibodies, which can recognize antigens expressed in follicular and interdigitating dendritic cells. Microscopically, IDCS has large fusiform spindle cells with indistinct cell borders that usually form a storiform or whorled growth pattern. The shape of the nucleoli is oval with finely dispersed chromatin. The nucleoli are small but prominent and sometimes multinucleated [12]. IDCS is characterized by positive staining for S-100, vimentin, HLA-DR, or CD68 [3], and negative staining for CD-1*α*, CD21, CD35, CD3, or CD20 [3, 13]. In our case, IDCS was also confirmed by positive staining for S-100, but not for CD-1*α*.

Many diseases are included in the differential diagnosis of extranodal interdigitating sarcoma in the nasal cavity: sarcomatoid carcinoma, melanoma, malignant peripheral nerve sheath tumor, atypical fibroxanthoma, malignant fibrous histiocytoma, rhabdomyosarcoma, and leiomyosarcoma [12]. These neoplasms usually lack spindle-shaped cells and whorled growth patterns. S-100 is a very specific marker for IDCS, but malignant melanoma and malignant peripheral nerve sheath tumors are also positive for S-100. CD68 and CD45 are negative in these two tumors [14]. CD-1*α*-positive status can be used to diagnose follicular dendritic cell sarcoma, rhabdomyosarcoma, or leiomyosarcoma [12].

Usual treatments for IDCS consist of surgical excision, systemic chemotherapy, or radiotherapy. Surgical resection is the mainstay of treatment in patients with localized disease. Radiation therapy, alone or with multiagent chemotherapy, has been used in several cases [3]. However, no therapeutic approach has demonstrated consistent clinical efficacy, and the role of chemotherapy or radiotherapy has not been clearly elucidated. Previously, adjuvant radiotherapy was found to prolong disease free states [9, 15], but adjuvant chemotherapy has not consistently shown good results. Therefore, adjuvant radiotherapy after complete surgical excision was considered in our case, and this combination has shown favorable results. 

FDCS, a different type of dendritic cell sarcoma, has poor prognostic factors such as intra-abdominal location, size greater than 6 cm, mitotic count greater than 5/10 high-powered fields, coagulative necrosis, significant nuclear pleomorphism, or lack of adjuvant therapy [9]. Compared with FDCS, IDCS behaves more aggressively because it is unresponsive to conventional therapy and spreads easily [2]. However, our case had a more favorable prognosis because the location of the tumor was confined to the nasal cavity without lymph node enlargement, and complete surgical excision was accomplished with adjuvant radiotherapy. If more cases of IDCS were available for analysis, additional research on the prognostic factors and treatment modalities according to age, size, location, or lymph node involvement would be elaborated in detail.

## 4. Conclusion

IDCS is an extremely unusual neoplasm that affects the lymphatic system as well as extranodal sites. Here, we presented a rare case of IDCS in the nasal cavity, which has been successfully treated with combined treatment of endoscopic sinus surgery and adjuvant radiotherapy. Although the incidence of IDCS in the nasal cavity is low, our case suggests that extranodal IDCS should be considered in the differential diagnosis of nasal cavity masses in the head and neck that are confirmed by immunohistochemistry.
